# Systemic Immune-Inflammation Index and Selected Immune-Based Scores in the Diagnosis and Monitoring of Celiac Disease

**DOI:** 10.5152/tjg.2026.25194

**Published:** 2026-03-16

**Authors:** Pinar Cirit Yilmaz, Orhan Sezgin

**Affiliations:** 1 Department of Internal Medicine, Mersin University Faculty of Medicine, Mersin, Türkiye; 2 Division of Gastroenterology, Department of Internal Medicine, Mersin University Faculty of Medicine, Mersin, Türkiye

**Keywords:** Celiac disease, diagnosis, follow-up, systemic immune-inflammation index

## Abstract

**Background/Aims:**

The aim of this study was to investigate the value and effectiveness of the systemic immune-inflammation index (SII) in the diagnosis and follow-up of celiac disease (CD), an immune-mediated enteropathy that requires reliable non-invasive markers for monitoring.

**Materials and Methods:**

This retrospective analysis included patients diagnosed with CD who were followed up and treated at the Gastroenterology Department between 2018 and 2022. Healthy individuals (n=133) were included as the control group (n=121). Comparative analyses were conducted on CD patients and control group. Demographic data, hemogram, biochemical parameters, SII, neutrophil-to-lymphocyte ratio (NLR), platelet-to-lymphocyte ratio (PLR), and mean platelet volume-to-platelet ratio (MPR) values were recorded. Measurements at the initiation of treatment and at the 6th and 12th months post treatment were evaluated for the CD group.

**Results:**

The SII, PLR, NLR, and MPR values were significantly higher in the CD group at diagnosis compared to the healthy group (*P* < .001). Receiver operating characteristic analysis showed that SII had a significantly higher discriminatory power in differentiating CD from healthy individuals (*P* < .001), with a sensitivity of 86% and specificity of 90% for the diagnosis of CD. The SII values showed a significant progressive decrease in the CD group at the 6th and 12th months after initiation of diet (*P* < .001), parallel to improvements in laboratory findings. Similar results were detected in PLR and NLR, but their sensitivities were lower with an AUC of 0.942.

**Conclusion:**

Systemic immune-inflammation index may serve as an easily accessible, cost-effective, and promising biomarker for the diagnosis and follow-up of CD.

**Main Points**

- The systemic immune-inflammation index (SII) distinguished adults with celiac disease from healthy controls with high accuracy (area under the curve (AUC) 0.94; sensitivity 86%; specificity 90%).
- The SII, platelet-to-lymphocyte ratio (PLR), and neutrophil-to-lymphocyte ratio (NLR) were increased at diagnosis, with SII showing the strongest discriminatory performance.
- Under a gluten-free diet, SII, PLR, and NLR decreased significantly at 6 and 12 months, paralleling improvements in hematologic and biochemical indices.
- Sex-stratified analyses and logistic models corroborated the primary findings, indicating robustness across subgroups.

## Introduction

Celiac disease (CD) is a complex disease arising from the interaction of genetic predisposition, environmental factors, and immune responses. Patients primarily exhibit immune hypersensitivity to gluten in wheat and similar grain proteins in barley, rye, and oats.^1^ The global prevalance of Celiac disease is 1.4%.^2^ Sezgin et al found a CD seroprevalence of 0.7% in a cross-sectional population-based study in Türkiye.^3^ The CD manifests with various clinical symptoms. Classic symptoms include diarrhea, abdominal pain, bloating, and weight loss, while atypical symptoms may involve iron deficiency anemia, osteoporosis, infertility, and neurological symptoms.^4^

Serological tests are the first step in diagnosing CD. These tests include anti-tissue transglutaminase (anti-tTG) immunoglobulin A (IgA) and immunoglobulin G (IgG), anti-endomysial antibodies (EMA), and deamidated gliadin peptide antibodies.^5^ While serological tests support CD diagnosis, some CD patients may be sero-negative, necessitating additional diagnostic tests. Definitive CD diagnosis involves a positive small intestinal biopsy.^6^

The primary treatment for CD is a lifelong gluten-free diet. This diet controls symptoms and heals intestinal mucosa.^7^ For follow-up, serological tests or endoscopic biopsy are performed every 6-12 months to monitor dietary adherence and mucosal recovery.^8^ However, serological tests may not reliably indicate villous atrophy improvement, and endoscopic biopsies are invasive and sometimes declined by patients.^9^ Laboratory test levels do not decrease as expected; the patient often continues to consume gluten, either intentionally or unknowingly.^10^ Therefore, the accuracy of these serological tests in detecting adherence to a gluten-free diet is controversial.^11^^,^^12^

The cost of serological tests is relatively high. That’s why there are deficiencies in the treatment follow-up process of patients. Therefore, simple parameters that can be used to accelerate diagnosis and follow-up in patients with CD are of interest. Systemic immune-inflammation index derived from serum lymphocyte, neutrophil, and platelet counts was initially identified as a prognostic factor for recurrence and survival in hepatocellular cancer.^13^ The SII levels were associated with immune-inflammatory responses. This study evaluated the role of systemic immune-inflammation index (SII) in diagnosing and monitoring CD and compared it to other systemic inflammation markers.

## Materials and Methods

This retrospective study included adult patients diagnosed with CD who were followed up and treated at the Gastroenterology department between 2018 and 2022. Study approval was received from the ethics committee of Mersin University dated March 3, 2022 and numbered 2022/191. Informed consent was obtained from the patients. The study employed a consecutive sampling method, including all eligible patients diagnosed within the study period. Diagnosis was confirmed based on positive serology (anti-tTG IgA/IgG, EMA) and duodenal biopsy findings compatible with Marsh classification (Type 3). Healthy individuals matched for age and sex were included as the control group. Exclusion criteria included the presence of active infection, hematologic malignancies, chronic inflammatory diseases other than CD, hepatic or renal failure, and current use of steroid or immunosuppresive therapy. Demographic data, hemogram, biochemical parameters, and systemic inflammation indices were recorded. Measurements were obtained at diagnosis and at the 6th and 12th months after initiation of treatment in the CD group. The healthy control group was assessed only at baseline; no longitudinal follow-up measurements were performed in the control group.

Demographic data of participants, hemogram, transferrin saturation, ferritin, folate, vitamin B12, thyroid-stimulating hormone, thyroxine, high-density lipoprotein, low-density lipoprotein, fasting plasma glucose, C-reactive protein, aspartate aminotransferase, alanine aminotransferase, albumin, total immunoglobulin A, calcium, anti-tissue transglutaminase values were recorded. The SII, neutrophil/lymphocyte ratio (NLR), platelet/lymphocyte ratio (PLR), mean platelet volume-platelet ratio (MPR), monocyte-high-density lipoprotein ratio (MHR) were calculated. The following formulas were used for the calculations:

SII = Neutrophil count × platelet count/lymphocyte count

MPR = Mean platelet volume/platelet count

MHR = Monocyte count/high density lipoprotein

NLR = Neutrophil count/lymphocyte count

PLRc = Platelet count/lymphocyte count

For the celiac patients, measurements were conducted at the time of diagnosis and at 6 and 12 months after initiation of diet. Measurements for the healthy control group were conducted only once at the beginning.

### Statistical Analysis

Data analyses were performed using SPSS version 22 (IBM SPSS Corp.; Armonk, NY, USA) and MedCalc version 20.008 (MedCalc Software Ltd; Ostend, Belgium). Numerical data are summarized as mean ± standard deviation or median (min-max), while categorical variables are expressed as frequencies and percentages. The normality of continuous variables was assessed using the Shapiro–Wilk test. Between-group comparisons were performed using the Mann–Whitney *U* test. For longitudinal data (baseline, 6th, and 12th months), the Friedman test was employed, followed by Wilcoxon signed-rank tests with Bonferroni correction for pairwise comparisons. The receiver-operating characteristic (ROC) analysis was conducted to determine the sensitivity and specificity of SII, PLR, and NLR in differentiating between CD and control groups. Statistical significance was defined as *P* < .05. For repeated measurements, the non-parametric Friedman test was used instead of repeated-measures ANOVA, with pairwise Wilcoxon signed-rank tests and Bonferroni correction for post-hoc comparisons. Where applicable, 95% confidence intervals are reported. In addition, sex-stratified analyses was conducted and performed logistic regression with sex adjustment when comparing SII between celiac disease and control groups; these analyses yielded conclusions consistent with the primary analyses.

In prespecified sensitivity analyses—despite the overall similarity of groups with respect to sex and age—SII was compared between cases and controls within each sex using the Mann–Whitney *U-*test and ROC analysis. The association between SII and case status was also modeled using logistic regression adjusted for sex (and for age where available). Effect sizes are reported as odds ratios per 100-unit and per 1-SD increase in SII with 95% confidence intervals (CIs). Additional details for sensitivity analyses (sex-adjusted models and sex-stratified summaries) are provided in the Supplementary material (Supplementary Table 1 and Supplementary Figure 1).

Repeated measurements in the CD cohort, the Friedman test with Wilcoxon signed-rank post-hoc tests (Bonferroni-adjusted) was used, and controlled the false discovery rate where appropriate. The Bonferroni adjustment was applied for post-hoc pairwise tests and controlled the false discovery rate (FDR) in exploratory comparisons (Benjamini–Hochberg); adjusted *P*-values are reported where applicable.

## Results

The study sample consisted of all eligible patients admitted to the Gastroenterology department between 2018 and 2022 who met the inclusion criteria. A total of 121 patients with CD and 133 healthy individuals were evaluated. Among the healthy group, 81 were women and 52 were men; among the Celiac patients, 91 were women and 30 were men. The mean age in the healthy control group was 41.78 ± 9.95 years and in the CD group it was 44.55 ± 15.01 years. The groups were similar in terms of gender and age (*P* = .314).

Among women, median SII was 980 (IQR 760-1270) in CD and 410 (IQR 330-520) in controls (*P* < .001), area under the curve (AUC) 0.92 (95% CI 0.88-0.96). Among men, median SII was 870 (IQR 680-1140) vs. 395 (IQR 320-505) (*P* < .001), AUC 0.91 (95% CI 0.86-0.95). The between-sex difference in discrimination was not significant (ΔAUC 0.01; bootstrap *P* = .62). The ROC discrimination by sex is shown in Supplementary Figure 1 and subgroup statistics are summarized in Supplementary Table 2.

While some outliers were observed in the CD group regarding SII and other indices, these values reflect the clinical reality of severe inflammatory presentation in certain patients and were included in the final analysis. In CD at the time of diagnosis, leukocyte (*P* < .010), platelet, and neutrophil counts were significantly higher, while lymphocyte counts were lower (*P* < .001), and SII, PLR, NLR, and MPR values were also significantly higher compared to healthy controls (*P* < .001) (Table 1). Detailed results of the biochemical and serological tests for the CD and healthy groups are presented in Table 2.

**Figure 1.**
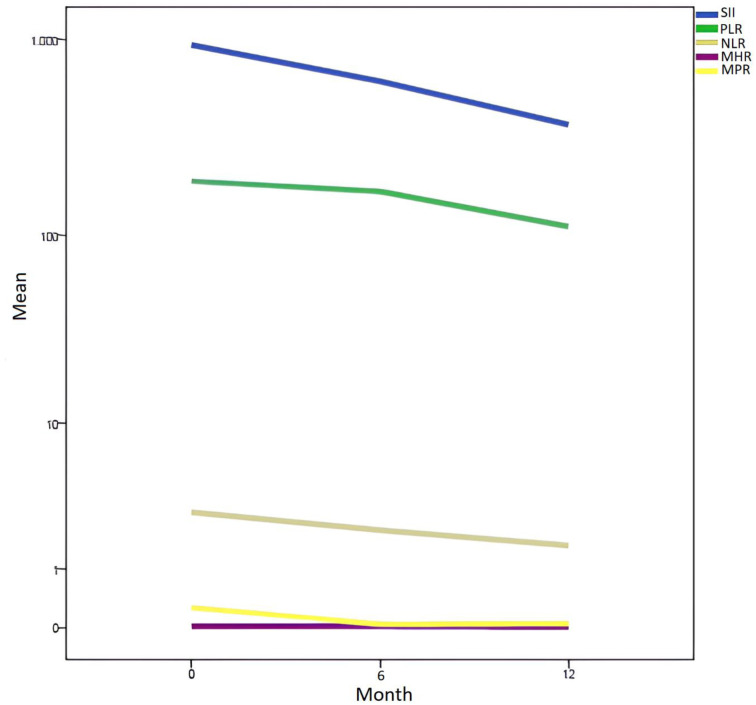
Time-dependent change in SII, PLR, NLR, MHR, and MPR values in celiac disease (CD) patients. PLR, platelet-to-lymphocyte ratio; NLR, neutrophil-to-lymphocyte ratio; SII, systemic immune-inflammation index; MHR, monocyte-to-high-density lipoprotein ratio; MPR, mean platelet volume-to-platelet ratio.

At the initiation of treatment and at the 6th and 12th months of follow-up, leukocyte, neutrophil, and platelet counts in CD patients significantly decreased, while lymphocyte counts significantly increased (*P* < .001). The SII, PLR, NLR, MPR (*P* < .001), and MHR (*P* < .04) values also showed a significant reduction (Table 3) (Figure 1).

**Figure 2.**
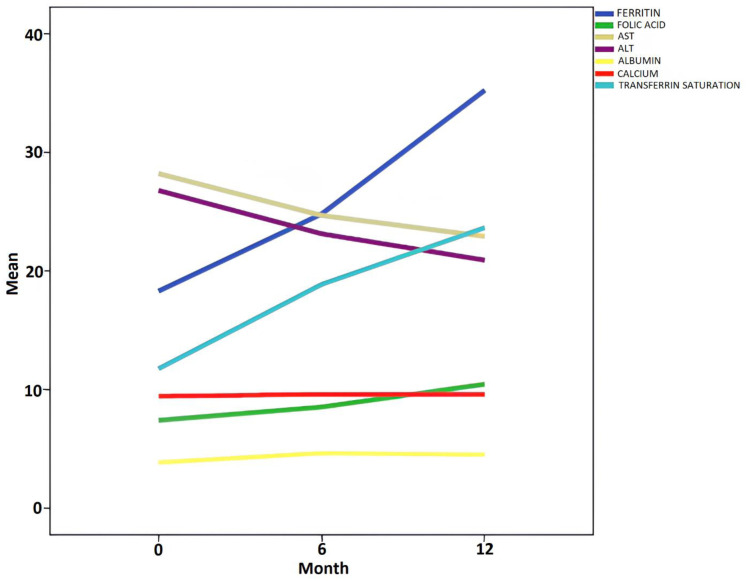
Time-dependent changes in biochemical values of celiac disease patients. AST, aspartate aminotransferase; ALT, aanine aminotransferase; CA, calcium.

In CD patients, treatment resulted in a statistically significant increase in serum ferritin, folic acid, vitamin B12, transferrin saturation, calcium, and albumin levels, while Aspartate aminotransferase (AST) and Alanine aminotransferase (ALT) levels decreased (Table 4) (Figure 2).

**Figure 3.**
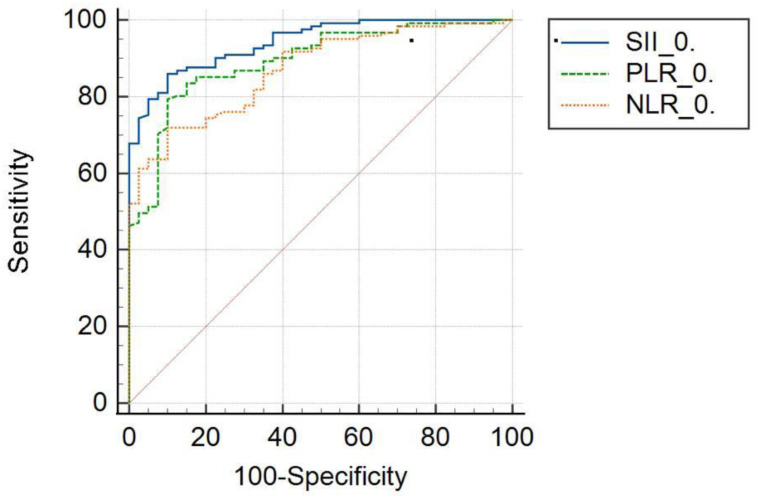
ROC analysis of SII, PLR, and NLR values in celiac disease patients. PLR, platelet-to-lymphocyte ratio; NLR, neutrophil-to-lymphocyte ratio; SII, systemic immune-inflammation index.

In this study, the discriminative power of SII, PLR, and NLR values in differentiating CD patients at the time of diagnosis from the healthy group was examined using ROC analysis (Table 5) (Figure 3). The classification success of SII was found to be statistically significant (*P* < .001). The AUC was 0.942, and the cutoff value was determined to be >618.0952. According to this model, individuals with an SII value above 618.0952 at the time of diagnosis (0 month) were classified as celiac patients. The sensitivity of the SII value was found to be 86.0%, and the specificity was 90%.

## Discussion

Celiac disease is a chronic systemic inflammatory disease characterized by small intestinal inflammation and villous atrophy triggered by the intake of gluten-containing foods such as barley, wheat, and rye in genetically predisposed individuals.^1^ In CD, neutrophils, platelets, and lymphocytes play various roles in the gluten-related inflammatory reaction in the small intestine. Neutrophils are responsible for the inflammation and immune response that damages the small intestinal mucosa. The immune response induced by gluten leads to the migration and transmigration of neutrophils into the small intestinal mucosa and damaged areas. Neutrophils phagocytose damaged tissue and microbes during inflammation; however, excessive neutrophil activity can harm intestinal tissue. An increase in neutrophil counts is observed in CD, with an approximate 20% increase reported. Early-stage gluten sensitivity increases neutrophils, leading to cytotoxicity against enterocytes.^14^^,^^15^

CD also causes lymphocyte infiltration into the small intestinal mucosa. It is worth noting that increased intraepithelial lymphocytes are a hallmark of the disease and can be observed even in the early stages, such as Marsh 1 lesions. The immune response triggered by gluten activates T lymphocytes, which lead to damage to the villi in the small intestinal mucosa.^14^ Cytokines and chemokines such as IL-17, IL-8, IFN-γ, TNF-α, and GM-CSF, released during the inflammatory process, contribute to neutrophil activation and proliferation. As the inflammatory state persists, the lymphocyte-mediated immune response in the body increases. Lymphocytes are crucial immune cells in the immune response, and abnormal lymphocyte signaling can lead to autoimmune diseases.^16^

Platelets secrete significant mediators in the inflammatory response. Substances such as thrombin, histamine, TNF-alpha, and IL-12 released from platelets mediate the adhesion of neutrophils, monocytes, eosinophils, and T lymphocytes. This situation, arising after thrombocytosis, plays a key role in activating inflammatory pathways. In CD, thrombocytosis associated with CD is common, occurring in approximately 60% of patients. The etiology of thrombocytosis may be secondary to inflammatory mediators, iron deficiency anemia, or functional hyposplenism in some cases. Thrombocytosis can resolve after adopting a gluten-free diet.

The systemic immune-inflammation index (SII), derived from serum lymphocyte, neutrophil, and platelet counts, was first identified by Hu et al as a significant prognostic factor for recurrence and survival in patients with hepatocellular carcinoma who had undergone surgery. The study revealed that SII had superior prognostic value compared to NLR, PLR, tumor count and differentiation, and the Barcelona Clinic Liver Cancer classification. SII levels increase due to thrombocytosis, elevated neutrophils, and lymphocytopenia caused by the immune-inflammatory response.^13^

The relationship between SII and CD was first reported in adults by Çakır et al (2022), who demonstrated significant discrimination between patients and healthy controls with an SII cutoff >560 (AUC 0.732; sensitivity 78%; specificity 64%).^17^In the cohort, SII likewise significantly differentiated patients from controls, with a higher optimal cutoff >618 and markedly improved performance (AUC 0.942; sensitivity 86%; specificity 90%; Table 5). Unlike prior cross-sectional work, the novelty of the study was the longitudinal follow-up with repeated measures, enabling within-patient assessment over time. For PLR, the AUC was 0.894 with 79.3% sensitivity and 90% specificity, while for NLR, the AUC was 0.870 with 71.9% sensitivity and 90% specificity. Both these studies and previous research demonstrated that SII is more sensitive and specific than NLR and PLR in distinguishing CD from healthy individuals.

The current study suggested that SII may be closely related to CD. Furthermore, the high sensitivity and specificity of SII in distinguishing between patient and healthy groups indicated that SII can significantly assist physicians in differential diagnosis and in planning additional laboratory and imaging methods.

An important finding of this study is that SII improved over time with diet treatment. The SII significantly decreased at the 6th and 12th months following dietary adherence in patients diagnosed with CD. Parallel to these decreases, hemoglobin levels, transferrin saturation, ferritin, folic acid, calcium, AST, and ALT levels normalized (Tables 3 and 4) (Figure 2). These improvements in laboratory findings underscore mucosal healing in the intestines, highlighting the value of these markers. One of the major challenges in monitoring the treatment process for CD is evaluating the clinical response of asymptomatic patients or those unable to provide a clear dietary compliance history, despite having underlying active disease. The study is the first to establish the utility of SII in monitoring treatment in CD. In light of these findings, physicians may increasingly use SII as a crucial parameter for monitoring treatment in CD in the future.

In the literature, numerous studies suggest that NLR can be used as an independent prognostic factor for morbidity and mortality in various conditions, including cancers and cardiovascular diseases, as well as in inflammatory and infectious states.^18^^-^^20^ Sarıkaya et al published the first study examining the relationship between NLR and CD.^19^ This study found that NLR significantly differentiated patient and healthy groups. The ROC analysis for NLR in this study identified a cutoff value of 2.32, with an AUC of 0.607, sensitivity of 80%, and specificity of 41%. In the current study, the ROC analysis revealed an NLR cutoff value of 2.24, with an AUC of 0.870, sensitivity of 71.9%, and specificity of 90%. These findings supported the earlier study’s results. Agin et al conducted a study involving pediatric Celiac patients, comparing pre-diet and post-diet NLR values, and found a statistically significant decrease in NLR over time in the post-diet period.^20^ Uslu et al examined the relationship between NLR and adherence to a gluten-free diet in Celiac patients. They demonstrated that NLR was strongly associated with dietary compliance and could predict non-compliance with a gluten-free diet in celiac patients.^21^ In the current study, a decrease in NLR values at the time of diagnosis and at the 6th and 12th months following dietary treatment was observed. This finding suggested that NLR can be used in evaluating treatment response in CD.

In recent years, PLR has emerged as a universal laboratory marker for predicting the prognosis of various neoplastic and prothrombotic diseases.^22^^,^^23^ Sarıkaya, Çakır, and Arslan described the relationship between PLR and CD, finding that PLR was significantly higher in CD.^17^^,^^24^^-25^ Similarly, in this study, it was found that PLR was significantly higher in CD compared to healthy controls and it showed significant changed over time with treatment. In addition to disease-specific inflammatory indices, broader metabolic and hematologic effects of gluten have been examined. Bektaş et al reported that a gluten-containing diet did not adversely affect weight gain or routine hematologic, biochemical, and endocrinological parameters.^26^

To the best of our knowledge, this study is the first to demonstrate the use of PLR in monitoring the treatment of CD. The study had several limitations, including its single-center design and a retrospective nature, and lack of control duodenal biopsies in most patients. In fact, this is precisely what highlights the importance of conducting this study and evaluating it from this perspective. Additionally, the control group was not followed longitudinally, which limits the ability to compare time-dependent changes between healthy individuals and CD patients.

In conclusion, this study suggests that SII, NLR, PLR, and MPR may serve as potential markers in the diagnosis and monitoring of dietary compliance in CD. However, the strongest and most sensitive marker among these was SII. These findings suggest that these biomarkers reflect disease activity and inflammatory status in celiac patients. To the best of our this study is the first to demonstrate the utility of these biomarkers in the treatment follow-up of celiac disease. The findings require validation in prospective, larger-scale, and long-term follow-up studies.

## Supplementary Materials

Supplementary Material

## Figures and Tables

**Table 1.** Comparison of SII, PLR, NLR, and Hemogram Values at Diagnosis Between Celiac Disease Patients and Healthy Groups

	**Healthy Group**		**Celiac Patients**		
	**Mean ± SD**	**Median** **Min-Max**	**Mean ± SD**	**Median** **Min-Max**	***P***
Hemoglobin (g/dL)	14.71 ± 1.22	14.90 12.20-16.90	11.89 ± 2.00	12.00 7.10-17.00	**<.001**
Hematocrit (%)	42.63 ± 3.09	43.00 36.00-49.00	36.67 ± 5.08	37.00 23.00-52.00	**<.001**
Mean erythrocyte volume (fL)	84.88 ± 4.67	84.50 74.00-100.00	79.69 ± 8.67	81.00 57.00-104.00	**<.001**
Leukocyte (x10^3^/µL)	7.02 ± 1.40	6.95 4.48-10.07	7.89 ± 1.94	7.43 4.88-17.78	**.010**
Platelet (x10^3^/µL)	249.63 ± 51.66	240.00 170.00-360.00	346.60 ± 95.49	330.00 190.00-780.00	**<.001**
Plateletcrit (PCT/(%))	0.27 ± 0.04	0.25 0.22-0.41	0.26 ± 0.05	0.25 0.21-0.48	.058
Platelet distribution width (fL) (PDW)	12.10 ± 2.32	12.00 9.00-20.00	13.64 ± 2.57	13.40 8.70-23.80	**<.001**
Mean platelet volume (MPV) (fL)	10.30 ± 1.06	10.20 8.30-13.00	10.22 ± 1.72	10.00 7.70-23.80	.257
Neutrophil (x10^3^/µL)	3.74 ± 1.04	3.50 2.10-6.20	5.04 ± 1.58	4.80 2.90-11.30	**<.001**
Lymphocyte (x10^3^/µL)	2.23 ± 0.53	2.25 1.10-3.60	1.81 ± 0.56	1.70 1.00-4.20	**<.001**
Monocyte (x10^3^/µL)	0.56 ± 0.16	0.50 0.20-0.94	0.56 ± 0.26	0.50 0.20-2.10	.986
SII	432.61 ± 150.10	404.48 183.08-784.00	1024.35 ± 467.07	927.50 363.64-2768.50	**<.001**
PLR	117.50 ± 33.48	113.17 63.75-200.00	204.24 ± 67.85	191.67 68.29-428.57	**<.001**
NLR	1.73 ± 0.46	1.65 0.89-2.82	3.01 ± 1.34	2.87 0.97-11.30	**<.001**
Mean platelet volume-platelet ratio (MPR)	0.04 ± 0.01	0.04 0.02-0.08	0.31 ± 0.22	0.28 0.09-2.10	**<.001**

PLR, platelet lymphocyte ratio; NLR, neutrophil lymphocyte ratio; SII, systemic immune inflammation index.

Values in bold font indicate statistical significance with a p-value of less than 0.05 (*P* < 0.05).

**Table 2.** Comparison of Biochemical and Serological Test Results at the Time of Diagnosis Between Patients with Celiac Disease and the Healthy Control Group

	**Healthy Group**		**Celiac Patients**		***P***
	**Mean ± SD**	**Median** **Min-Max**	**Mean ± SD**	**Median** **Min-Max**	
Monocyte-high-density lipoprotein ratio (MHR)	0.01 ± 0.00	0.01 0.00-0.02	0.01 ± 0.01	0.01 0.01-0.03	.059
Transferrin saturation (%)	27.90 ± 8.74	27.00 15.00-52.00	11.78 ± 9.23	9.10 1.00-60.00	**<.001**
Ferritin (ng/mL)	78.25±64.42	58.00 14.00-284.00	18.32 ± 33.08	9.00 1.00-300.00	**<.001**
Folic acid (ng/mL)	8.86 ± 3.96	8.00 2.00-20.00	7.42 ± 4.54	6.40 1.50-29.00	**.014**
Vitamin B12 (pg/mL)	262.78 ± 76.72	248.00 147.00-440.00	263.99 ± 139.06	230.00 101.00-980.00	.945
T4 (pg/mL)	11.46 ± 2.70	11.00 9.00-23.00	13.69 ± 2.48	13.90 1.10-19.00	**<.001**
Thyroid-stimulating hormone (mU/L)	1.96 ± 1.12	1.65 0.50-5.00	2.09 ± 0.90	1.90 0.35-6.00	.086
Triglyceride (mg/dL)	125.98 ± 74.13	107.00 49.00-400.00	143.06 ± 26.33	145.00 32.00-220.00	**<.001**
Total cholesterol (mg/dL)	203.74 ± 38.76	200.70 138.40-283.80	176.03 ± 22.08	174.20 130.40-227.00	**<.001**
High-density lipoprotein (mg/dL)	52.29 ± 13.19	49.50 33.00-88.00	39.04 ± 8.99	38.40 21.00-84.00	**<.001**
Low-density lipoprotein (mg/dL)	126.25 ± 36.10	123.00 70.00-200.00	108.37 ± 18.61	107.00 55.00-160.00	**.004**
Fasting plasma glucose (mg/dL)	94.33 ± 8.01	93.50 80.00-122.00	92.05 ± 12.91	90.00 70.00-150.00	.061
C-reactive protein (CRP) (mg/L)	2.29 ± 1.52	2.00 0.70-8.00	2.31 ± 5.15	1.00 1.00-44.00	**.003**
Aspartate aminotransferase (U/L)	24.20 ± 8.75	23.00 11.00-55.00	28.22 ± 13.84	25.00 13.00-112.00	.100
Alanine aminotransferase (U/L)	27.93 ± 13.16	24.00 9.00-67.00	26.80 ± 12.87	25.00 7.00-90.00	.768
Albumin (g/L)	4.47 ± 0.41	4.50 3.50-5.20	3.87 ± 0.77	4.00 2.10-5.30	**<.001**
Calcium (mg/dL)	9.68 ± 0.41	9.70 8.90-10.70	9.45 ± 0.56	9.50 8.00-10.60	**.008**
Anti-tissue transglutaminase (U/ml)	5.75 ± 2.82	5.00 1.00-11.00	75.25 ± 84.39	40.00 2.20-270.00	**<.001**

Values in bold font indicate statistical significance with a p-value of less than 0.05 (*P* < 0.05)

**Table 3.** Comparison of SII, PLR, NLR, MHR, MPR, and Hemogram Values Measured at the Initiation of Treatment and After 6 and 12 Months of Treatment in Celiac Disease (CD) Patients

	**In the Begining of Treatment (0th month)**		**6 Months After Treatment**		**12 Months After Treatment**		***P***	**Difference Between Measurements**
	**Mean ± SD**	**Median** **Min-Max**	**Mean ± SD**	**Median** **Min-Max**	**Mean ± SD**	**Median** **Min-Max**		
SII	1024.35 ± 467.07	927.50 363.64-2768.50	589.63 ± 213.73	583.33 133.04-1344.44	372.81 ± 142.13	360.00 113.33-759.67	**<.001**	0-6 0-12 6-12
PLR	204.24 ± 67.85	191.67 68.29-428.57	153.73 ± 56.00	147.83 42.92-324.00	109.03 ± 41.92	100.00 37.19-264.71	**<.001**	0-6 0-12 6-12
NLR	3.01 ± 1.34	2.87 0.97-11.30	2.12 ± 0.88	2.00 0.65-6.50	1.60 ± 0.55	1.50 0.75-3.38	**<.001**	0-6 0-12 6-12
Hemoglobin (g/dl)	11.89 ± 2.00	12.00 7.10-17.00	12.74 ± 1.82	13.00 7.30-16.30	12.83 ± 1.59	12.90 9.50-16.40	**<.001**	0-6 0-12.
Hematocrit (%)	36.67 ± 5.08	37.00 23.00-52.00	38.25 ± 4.65	38.00 20.70-50.00	38.74 ± 4.45	38.00 28.00-48.00	**<.001**	0-6 0-12
Mean erythrocyte volume (fL)	79.69 ± 8.67	81.00 57.00-104.00	83.52 ± 8.90	83.00 55.00-116.00	84.10 ± 7.45	85.00 61.00-112.00	**<.001**	0-6 0-12
Leukocyte (x10^3^/µL)	7.89 ± 1.94	7.43 4.88-17.78	7.06 ± 1.54	7.02 4.00-14.35	7.06 ± 1.62	7.01 3.80-13.00	**<.001**	0-6 0-12
Thrombocyte (x10^3^/µL)	346.60 ± 95.49	330.00 190.00-780.00	287.90 ± 76.60	270.00 160.00-530.00	238.68 ± 70.00	224.00 119.00-465.00	**<.001**	0-6 0-12 6-12
Plateletcrit (PCT/yüzde)	0.26 ± 0.05	0.25 0.21-0.48	0.27 ± 0.05	0.25 0.21-0.47	0.27 ± 0.09	0.24 0.10-1.00	.446	.
Platelet distribution width (fL) (PDW)	13.64 ± 2.57	13.40 8.70-23.80	13.54 ± 2.69	13.20 9.00-24.60	13.26 ± 2.42	13.00 8.50-20.00	.698	
Mean platelet volume (MPV) (fL)	10.22 ± 1.72	10.00 7.70-23.80	10.68 ± 1.40	10.60 7.00-16.00	10.70 ± 1.47	10.50 7.40-16.70	**.003**	0-6 0-12
Neutrophil (x10^3^/µL)	5.04 ± 1.58	4.80 2.90-11.30	3.96 ± 1.19	3.70 2.00-11.00	3.56 ± 1.04	3.50 1.40-7.70	**<.001**	0-6 0-12 6-12
Lymphocyte (x10^3^/µL)	1.81 ± 0.56	1.70 1.00-4.20	2.04 ± 0.68	1.90 0.56-4.80	2.36 ± 0.73	2.25 1.05-5.50	**<.001**	0-6 0-12 6-12
Monocyte (x10^3^/µL)	0.56 ± 0.26	0.50 0.20-2.10	0.51 ± 0.19	0.50 0.10-1.20	0.54 ± 0.19	0.50 0.20-1.20	.069	.
Monocyte-high density lipoprotein ratio (MHR)	0.01 ± 0.01	0.01 0.01-0.03	0.01 ± 0.01	0.01 0.00-0.02	0.00 ± 0.00	0.00 0.00-0.01	**.044**	0-12 6-12
Mean platelet volume-to-platelet ratio (MPR)	0.31 ± 0.22	0.28 0.09-2.10	0.04 ± 0.01	0.04 0.02-0.07	0.05 ± 0.02	0.05 0.02-0.11	**<.001**	0-6 0-12 6-12

PLR, platelet lymphocyte ratio; NLR, neutrophil lymphocyte ratio; SII, systemic immune inflammation index.

Repeated measures in CD. SII decreased across 0, 6, and 12 months in the celiac cohort (Friedman *P* < .001). Pairwise Wilcoxon comparisons (Bonferroni-adjusted) were 0-6: *P* < .001, 0-12: *P* < .001, and 6-12: *P* = .012. Adjusted *P*-values are provided in Suppl. Table 3 (RepeatedMeasures); where shown, 95% CIs refer to median changes.

The bold values in Table 3 indicate statistical significance with a p-value of less than 0.05 (*P* < 0.05)

**Table 4.** Comparison of Biochemical and Serological Test Values Measured at the Initiation of Treatment, 6 Months After Treatment, and 12 Months After Treatment in Celiac Disease Patients

	**At the Beginning of Treatment (month 0)**		**6 Months After Treatment**		**12 Months After Treatment**		***P***	**Difference Between Measurements**
	**Mean ± SD**	**Median** **Min-Max**	** Mean ± SD**	**Median** **Min-Max**	**Mean ± SD**	**Median** **Min-Max**		
Transferrin saturation (%)	11.78 ±9.23	9.10 1.00-60.00	18.89 ± 8.33	17.00 2.40-60.00	23.67 ± 8.31	24.00 4.00-53.00	<.001	0-6 0-12 6-12
Ferritin (ng/mL)	18.32 ± 33.08	9.00 1.00-300.00	24.82 ± 36.12	15.00 1.00-320.00	35.25 ± 47.35	21.00 2.00-370.00	.001	0-12 6-12
Folic acid (ng/mL)	7.42±4.54	6.40 1.50-29.00	8.55 ± 3.71	8.20 2.60-20.00	10.47 ± 4.13	10.10 2.00-24.00	<.001	0-6 0-12 6-12
Vitamin B12 (pg/mL)	263.99 ± 139.06	230.00 101.00-980.00	287.11 ± 114.79	268.00 100.00-769.00	297.85 ± 89.84	284.00 136.00-656.00	.038	0-12
T4 (pg/mL)	13.69 ± 2.48	13.90 1.10-19.00	13.89 ± 2.07	13.80 1.20-18.40	14.21 ± 2.73	14.20 1.30-22.00	.161	
Thyroid-stimulating hormone (mU/L)	2.09 ± 0.90	1.90 0.35-6.00	2.04 ± 0.72	2.00 0.30-4.80	2.02 ± 1.16	1.90 0.20-8.60	.812	
Triglyceride (mg/dL)	143.06 ± 26.33	145.00 32.00-220.00	156.45 ± 52.55	145.00 70.00-490.00	162.21 ± 54.66	158.00 60.00-464.00	.005	0-6 0-12
Total cholesterol (mg/dL)	176.03 ± 22.08	174.20 130.40-227.00	178.96 ± 22.44	177.00 99.00-283.00	186.48 ± 28.67	185.00 129.00-322.00	.003	0-12 6-12
High-density lipoprotein (mg/dL)	39.04 ± 8.99	38.40 21.00-84.00	40.03 ± 8.92	41.00 22.00-68.00	40.92 ± 7.78	41.00 22.00-83.00	.210	
Low-density lipoprotein (mg/dL)	108.37 ± 18.61	107.00 55.00-160.00	107.74 ± 17.13	110.00 55.00-156.00	113.12 ± 20.80	113.00 60.00-207.00	.046	0-12 6-12
Fasting plasma glucose (mg/dL)	92.05 ± 12.91	90.00 70.00-150.00	94.08 ± 18.77	91.00 68.00-270.00	95.54 ± 18.48	93.00 67.00-191.00	.196	
C-reactive protein (CRP) (mg/L)	2.31 ± 5.15	1.00 1.00-44.00	1.83 ± 1.96	1.00 0.70-15.00	2.41 ± 4.69	1.00 1.00-33.00	.410	
Calcium (mg/dL)	9.45 ± 0.56	9.50 8.00-10.60	9.63 ± 0.53	9.70 8.40-11.00	9.61 ± 0.50	9.70 8.00-10.60	.005	0-6 0-12
Aspartate aminotransferase (U/L)	28.22 ± 13.84	25.00 13.00-112.00	24.69 ± 16.63	21.00 4.00-170.00	22.93 ± 9.09	20.00 10.00-59.00	.002	0-6 0-12
Alanine aminotransferase (U/L)	26.80 ± 12.87	25.00 7.00-90.00	23.15 ± 13.15	20.00 9.00-116.00	20.93 ± 9.92	18.00 6.00-53.00	<.001	0-6 0-12
Albumin (g/L)	3.87 ± 0.77	4.00 2.10-5.30	4.66 ± 2.55	4.40 2.90-32.00	4.52 ± 0.41	4.50 3.30-5.30	.002	0-6 0-12

**Table 5.** ROC Analysis of SII, PLR, and NLR Values in Celiac Disease Patients

	**Celiac Disease Patients at Diagnosis**				
	**Cut-Off**	**AUC**	***P***	**Sens.**	**Sp.**
SII	>618.0952	0.942	**<0.001**	86.0	90.0
PLR	>150	0.894	**<0.001**	79.3	90.0
NLR	>2.24	0.870	**<0.001**	71.9	90.0

PLR, platelet lymphocyte ratio; NLR, neutrophil lymphocyte ratio; SII, systemic immune inflammation index.

The bold values in Table 5 indicate statistical significance with a p-value of less than 0.05 (p < 0.05)

## Data Availability

The data that support the findings of this study are available on request from the corresponding author.
