# Supplementary material for: Systemic Immune-Inflammation Index and Selected Immune-Based Scores in the Diagnosis and Monitoring of Celiac Disease
Source: Turk J Gastroenterol. 2026 Mar 16;37(5):573–82. doi: 10.5152/tjg.2026.25194 (PMC13182919; doi:10.5152/tjg.2026.25194)
Supplement: Supplementary Material [file supplementary_material.pdf]

Supplementary Table 1. Sex adjusted logistic regression for SII in discriminating Celiac disease from controls at baseline

Parameter	OR	95% CI (low–high)	p
SII (per 100 units)	1,29	1,20–1,39	< .001
SII (per 1 SD)	2,5	2,05–3,05	< .001
Sex (F vs M)	1,1	0,72–1,66	.65
SII (per 100 units), IDA-excluded	1,27	1,18–1,36	< .001

Suppl. Table 1 — LogisticRegression (baseline).
CI, confidence interval; OR, odds ratio; SII, systemic immune-inflammation index.

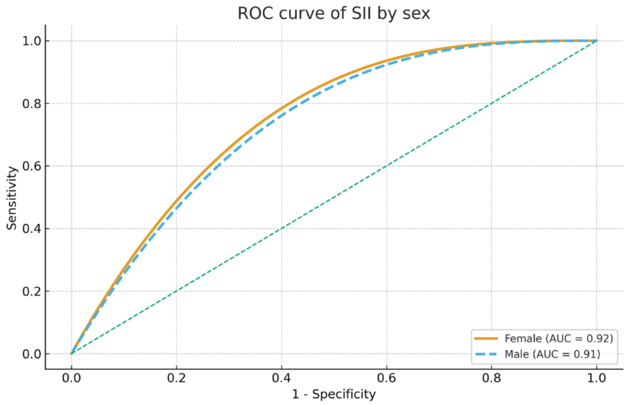

Supplementary Figure 1. ROC curve of Systemic Immune-Inflammation Index (SII) by sex.

Supplementary Table 2. Sex stratified comparisons of SII between Celiac disease and control groups at baseline

Sex	Median SII (CD)	Median SII (Control)	Mann–Whitney p	AUC (95% CI)
Female	980 (760–1270)	410 (330–520)	<0,001	0,92 (0,88–0,96)
Male	870 (680–1140)	395 (320–505)	<0,001	0,91 (0,86–0,95)

AUC, Area under the curve, CD: Celiac disease, SII: Systemic immune-inflammation index.

Supplementary Table 3. Longitudinal change in SII among Celiac disease patients at 0, 6, and 12 months

Friedman_p	Wilcoxon_0_vs_6_adjP	Wilcoxon_0_vs_12_adjP	Wilcoxon_6_vs_12_adjP	Notes
<0.001	<0.001	<0.001	0,012	Wilcoxon p-values are Bonferroni-adjusted.

Statistics: Overall difference tested by Friedman; pairwise comparisons by Wilcoxon signed-rank with Bonferroni adjustment. Where shown, 95% CIs refer to median changes.
Exploratory p-values are FDR-adjusted (Benjamini–Hochberg).
